# Support for Local Tobacco Policy in a Preemptive State

**DOI:** 10.3390/ijerph16183378

**Published:** 2019-09-12

**Authors:** Rebekah R Rhoades, Laura A Beebe, Nasir Mushtaq

**Affiliations:** Department of Biostatistics and Epidemiology, Hudson College of Public Health, University of Oklahoma Health Sciences Center, 801 NE 13th Street, Oklahoma City, OK 73104, USA

**Keywords:** tobacco control policies, social influence, secondhand smoke knowledge, community readiness, survey, Tobacco Nation

## Abstract

Policy at the local level is a critical component of comprehensive tobacco control programs. This study examined the relationships of individual and social factors with support for tobacco-related public policy using cross-sectional data (n = 4461) from adults participating in a statewide survey. Weighted multivariate, multinomial logistic regression examined associations between individual and social factors and support for tobacco-free city properties and support for limiting the number of stores that sell tobacco near schools. Oklahomans were more likely to favor policies that create tobacco-free city properties than policies that limit the number of stores that sell tobacco near schools. While non-smokers were most likely to favor both policies, support for both policies was greater than 50% among current smokers. Knowledge of secondhand smoke (SHS) exposure harm and female gender were predictors of support for both policies and among current, former, and never smokers. Rural-urban status was a predictor of support among former smokers and never smokers. Tobacco use among friends and family was only a predictor among never smokers’ support for limiting the sale of tobacco near schools. This study demonstrates that level of support differs by policy type, individual smoking status, as well as among subpopulations, and identifies critical elements in the theory of change for tobacco control programs.

## 1. Introduction

Oklahoma has one of the highest tobacco prevalence rates in the nation. According to 2017 BRFSS data, Oklahoma’s smoking prevalence was 20.1% compared to the national average of 14.0% [1,2]. Best practices, informed by years of research, have established the critical need for comprehensive state tobacco control programs that include local policy measures proven to reduce tobacco use and its associated morbidity and mortality [3,4,5,6,7,8,9]. 

“Oklahoma has been slow to experience statewide tobacco control and prevention policy wins that have been associated with reduced prevalence in other states. This is due in part to the powerful presence and influence the tobacco industry lobby has had in state government” [3,10,11]. Oklahoma laws governing clean indoor air and youth access include tobacco industry supported preemptive language. The state law related to clean indoor air includes exemptions for bars, restaurants, outdoor seating areas of restaurants, hotels, in-home child care centers when children are not present, workplaces with incidental public access, and allows for all workplaces to provide smoking rooms in which no work is performed. The law preempts municipalities from enacting laws more stringent than state law but does include clarifying language that states local municipalities have the discretion to prohibit smoking in or on property owned or operated by their own governing body [12]. State law related to preventing youth access to tobacco prohibits municipalities from enacting any law related to the sale, purchase, distribution, advertising, sampling, promotion, display, possession, licensing, or taxation of tobacco products that is stronger than state law [13]. According to the Centers For Disease Control and Prevention’s State Tobacco Activities Tracking and Evaluation (STATE) System, Oklahoma is one of eight states with preemptive language governing both smokefree indoor air and youth access. Other states include Michigan, North Carolina, Pennsylvania, South Dakota, Tennessee, Utah, and Washington [14]. The combination of state laws that do not fully protect Oklahomans, plus the inclusion of preemptive language, places Oklahoma communities at a deficit in terms of ability to protect their citizens and prevent tobacco use by implementing best practices and innovative approaches to tobacco control.

Studies have shown that local level tobacco control policies often lead to statewide adoption of policies [15]. Local policies that result from grassroots community efforts increase local awareness of tobacco control issues and build community readiness and support. Research has also demonstrated that when communities are limited in these efforts through preemption, health and social norm disparities are created between states where local authorities have the ability to adopt tobacco control policies and states where local authorities are preempted from enacting such policies [16,17]. As has been experienced in Oklahoma, once enacted, preemption is difficult to repeal, and can limit community-based tobacco control programming [18]. 

Additionally, Oklahoma is part of a band of 13 states that not only have a significantly higher smoking prevalence than the US average but also a rate that is higher than the smoking prevalence of many of the world’s most tobacco dependent countries. This region of states was identified by Truth Initiative^®^ and termed “Tobacco Nation” [19,20]. Truth Initiative^®^ also found that Tobacco Nation residents are less likely to be protected by policies that are known to impact the rate of tobacco use and reduce preventable death and disease caused by tobacco [21]. Protective policies include smokefree policies, tobacco prices and taxes, and policies that influence access to tobacco products. 

On 1 July 2015, the Tobacco Settlement Endowment Trust launched the five-year Healthy Living Program (TSET HLP) grant initiative in 63 of Oklahoma’s 77 counties, covering 94% of the state’s population. This statewide program aims to reduce cancer and cardiovascular disease by preventing and reducing tobacco use and obesity on a local level. The TSET HLP Community Survey is one component of the evaluation of the statewide program. It measures population-level change in knowledge, attitudes, and behaviors among adults living in the funded counties. Survey objectives included assessing support for two of the governmental policy strategies contained within defined programmatic outcomes. Due to preemption, the two proposed policies are narrower in scope than policy strategies available to non-preempted municipalities outside of Oklahoma. The only clean air related policy that local municipalities in Oklahoma can enact, outside of mirroring state law, is one that establishes their own properties, indoor and outdoor, as tobacco-free. The second policy, limiting the sale of tobacco near schools, is allowable under preemption only if accomplished through zoning procedures which are not covered by preemption in Oklahoma. 

The TSET HLP Community Survey offers a unique opportunity to examine community readiness to enact the policy changes included in the program’s defined outcomes. The Community Readiness Model posits that in order to move a population to implementation of public policy, the population must perceive tobacco use as a serious concern, they must be aware of local efforts addressing the issue of tobacco control and prevention, and they must have knowledge about issues related to tobacco use [22,23]. Additionally, belief in the harmfulness of secondhand smoke has been consistently associated with support for smokefree policies [5,24].

To assess support for tobacco-free properties and clean indoor air, respondents were asked the extent that they favored or opposed government policies that would establish tobacco-free city properties. To assess support for reducing access to tobacco, especially among youth, respondents were asked the extent they favored or opposed limiting the number of stores that sell tobacco near schools.

The purpose of this paper is to describe (1) the extent that tobacco use is perceived as a serious community problem; (2) level of awareness of community efforts to reduce tobacco use; (3) social influence related to tobacco; (4) knowledge of the harm related to secondhand smoke (SHS) exposure; and (5) other demographic factors are associated with policy support. 

This study showed that the majority of Oklahomans favor policies that establish city property as tobacco-free and that limit the sale of tobacco near schools. While non-smokers were most likely to favor each of the policies, support was also high among former smokers, and support for both policies was greater than 50% among current smokers. Female gender and knowledge of the harm associated with SHS exposure were consistent, strong predictors of support for both policies and among current, former, and never smokers, indicating a continued need for education about the adverse effects of exposure to SHS.

## 2. Materials and Methods

The study design is cross-sectional with two waves of data collection through a telephone survey of land line and cell phones. This paper includes baseline data from the first wave of data collection in 2016. The target population for the survey consisted of non-institutionalized adults residing in one of the 63 counties served by the TSET HLP. Random samples were used to complete surveys via landlines (*n* = 1097) and cell (*n* = 3364) telephone numbers, for a total sample size of 4461. A stratified sampling design was used to ensure that each of six pre-defined regions in Oklahoma was equally represented. The response rate was 10% (AAPOR RR1) [25]. Weighted estimates were calculated and adjusted for non-coverage and non-response, creating estimates more representative of the Oklahoma population residing in the HLP counties. All subjects gave their oral verbal informed consent for inclusion before they participated in the study. The study was approved by the Institutional Review Board of the University of Oklahoma Health Sciences Center (#6351) on 01/22/2016.

### 2.1. Outcome Measures

Participants were asked, “Do you favor, oppose, or neither favor nor oppose the following government policies?”

a Policies that prohibit tobacco use on city-owned properties.
b Policies that limit the number of stores that sell tobacco near schools.

A 5-point Likert measurement scale was used. Respondents who answered “strongly favor” or “somewhat favor” were categorized as favoring the policy. Those that answered “strongly oppose” or “somewhat oppose” were categorized as opposing the policy. A third category of those answering “neither favor nor oppose” was also used. 

### 2.2. Smoking Status

Current smoking status was determined by the answers to two questions. “Have you smoked at least 100 cigarettes in your life? And, “Do you now smoke cigarettes every day, some days, or not at all?” Current smokers were defined as those that had smoked at least 100 cigarettes in their life and now smoke every day or some days. Former smokers were defined as those that had smoked at least 100 cigarettes in their life and now do not smoke at all. Never smokers were defined as those that had not smoked at least 100 cigarettes in their life. 

### 2.3. Sociodemographic Factors

Six geographical regions of the state were included: Northwest, Northeast, Tulsa, Central, Southwest, and Southeast. These regions reflect those used in statewide surveillance efforts such as implementation of the Behavioral Risk Factor Surveillance System. Rural-urban status was assigned at the county level. The urban category includes the three most populous counties in the state and accounts for about 43% of the state’s population. The remaining TSET HLP counties were categorized as rural. Several sociodemographic measures were included, such as gender (female or male), age (18–34, 35–54, 55+), race/ethnicity (White, Black or African American, American Indian or Alaska Native, Hispanic, Multiracial), education (high school or less, some college or more), income (≤$30,000, $30,000–<$45,000, $45,000–<$60,000, $60,000–<$80,000, $80,000–<$100,000, $100,000–<$150,000, ≥$150,000) and marital status (married, widowed, divorced or separated, never married, other).

### 2.4. Social Influence

Respondents were asked the question “How many of the people that are important to you smoke or use other tobacco products?” Answer options included: none of them, less than half of them, about half of them, more than half of them, and all of them. Based on the distribution of responses and methods used in previously published research examining the influence of family and friends smoking, a dichotomous version of this variable was created—*none* and *any* [26,27]. Respondents that answered “none” were categorized as having none of the people important to them using tobacco. All other respondents were placed in the “any” category.

### 2.5. Knowledge, Perceptions, and Awareness

For this analysis, a knowledge summary score was created based on responses to four questions. Respondents’ scores ranged from 0 to 4 based on the number of correct responses (“Yes”) to the knowledge-based questions. “Don’t know” responses were combined with the “No” responses. The four knowledge questions reflect questions used in prior surveys and priorities within the state’s health communication campaign, *Tobacco Stops with Me*.

1 Does breathing smoke from other people’s cigarettes cause Sudden Infant Death Syndrome, or SIDS?
2 Does breathing smoke from other people’s cigarettes cause ear infections in children?
3 Does breathing smoke from other people’s cigarettes cause heart disease in adults?
4 Does breathing smoke from other people’s cigarettes cause asthma?

The summary score was used in the multivariate, multinomial analysis, and the mean score was reported among those who favor, oppose, and neither favor nor oppose each policy.

Respondents were asked the question “How serious of a problem is smoking and tobacco use in your community?” A 4-point Likert scale was used. Answer options included: not a serious problem, only a little serious, fairly serious, and very serious. A dichotomous version of this variable was created based on the distribution of responses and methods used in previous research assessing perceptions of tobacco use [28]. Due to small sample sizes for “not a serious problem” and “only a little serious” as well as no significant differences between these response options and the “fairly serious” option, we grouped these as one category. Those answering “very serious” were placed in one category and those answering “not a serious problem, only a little serious, and fairly serious” were placed in the other category.

Awareness of local, community efforts addressing the issue of tobacco use was also included as a measure. Awareness was measured by the item, “Are you aware of any programs, activities, services, or policies to decrease tobacco use or exposure to secondhand smoke in your community?” Answer options were “yes” and “no”. Some participants volunteered the answer “don’t know”. “Don’t know” responses were combined with “no” responses. 

### 2.6. Statistical Methods

Data were analyzed using SAS, version 9.4 (SAS Institute Inc., Cary, NC, USA). Weighted estimates with 95% confidence intervals for support of the two policies were calculated and stratified by covariates. Weighted multivariate, multinomial logistic regression models were constructed with three levels of responses to the policy questions: favor, neither favor nor oppose, and oppose. Oppose served as the reference category. Interaction was present by smoking status; thus, all models were stratified by three levels of smoking status: current smokers, former smokers and never smokers. All covariates were included as potential confounders in the models, using purposeful selection and based on previous research [29]. Adjusted odds ratios (OR) and 95% confidence intervals (CI) were reported. Assessment of multicollinearity included comparison of standard errors (univariate vs. multivariate models) obtained from the SAS SurveyLogistic procedure, evaluation of variance inflation factor (VIF), and Tolerance obtained from linear regression model. All showed no sign of multicollinearity effect. 

## 3. Results

### 3.1. Prevalence of Support

#### 3.1.1. Support for Tobacco-Free City Property Policies

Three-fourths (75.3%) of respondents favored policies that prohibit tobacco use on city property. As shown in Table 1, the prevalence of favoring policies that make city property tobacco-free was higher among females (81.5%) than males (68.8%); among residents residing in the Central region of the state (79.5%) than those residing in the Northwest region (69.6%); among urban residents (78.8%) than rural residents (72.2%); and among those with some college or more (78.0%) than those with a high school or less education (71.7%). 

Perceptions, social influence, and knowledge were also associated with favorable attitudes toward policies that prohibit tobacco use on city property. Prevalence was higher among respondents that perceived smoking and tobacco use as a “very serious” problem for people in their community (78.9%) than those who reported smoking and tobacco use as a “fairly serious/only a little serious/not a serious problem” within their community (71.6%); among respondents that reported that “none” of the people that were important to them use tobacco (82.3%) than those that reported that “any” of the important people in their lives use tobacco (73.0%); among those that correctly answered all four knowledge questions (84.4%) than those that answered two questions correctly (75.3%), those that answered one question correctly (67.8%) and those that answered none of the knowledge questions correctly (51.8%). Additionally, the mean knowledge score among those that favored the policy was significantly higher than those that “opposed” or “neither favored nor opposed the policy” (2.5 vs. 2.0, 1.9). 

By smoking status, prevalence of favorable attitudes toward policies that make city property tobacco-free was higher among never smokers (82%) than former smokers (72.7%) or current smokers (56.4%). Prevalence of reporting “neither favor nor oppose” the policy was higher among current smokers (17.2%) than former smokers (9.6%) and never smokers (7.7%). 

No meaningful differences were observed in the prevalence of favoring polices that make city property tobacco-free among race and ethnicity groups, age, income, marital status groups, and awareness of efforts in the community to decrease tobacco use and prevent secondhand smoke exposure. 

#### 3.1.2. Support for Policies that Limit the Number of Stores that Sell Tobacco Near Schools

Two-thirds (66.7%) of respondents favored policies that limit the number of stores that sell tobacco near schools. As shown in Table 2, favoring the policy was higher among females (72.7%) than males (60.6%); among those residing in the Central (most populous) region of the state (72.8%) than those residing in the Northwest region (56.9%), and those residing in the Southwest region (62.5%); and among urban residents (71.3%) compared to rural residents (62.9%). 

Favor for policies that limit the number of stores that sell tobacco near schools was lowest among those in the highest income group, more than $150,000 (60.5%). 

Social influence and knowledge were also associated with favorable attitudes toward policies that limit the number of stores that sell tobacco near schools. Support was higher among respondents that reported that “none” of the people that were important to them used tobacco (72.1%) than those that reported that “any” of the important people in their lives use tobacco (65.0%); among those that correctly answered all four knowledge questions (79.5%) than those that answered two questions correctly (68.4%), those that answered one question correctly (56.9%) and those that answered none of the knowledge questions correctly (41.5%). Additionally, the mean knowledge score among those that favored the policy was higher than those that “opposed” or “neither favored nor opposed” the policy (2.54 vs. 2.0, 2.01). 

By smoking status, prevalence of favorable attitudes toward policies that limit the number of stores that sell tobacco near schools was higher among never smokers (72.7%) than former smokers (61%) or current smokers (54.4%). Prevalence of reporting “neither favor nor oppose” the policy was higher among current smokers (17.9%) than never smokers (11.5%).

No meaningful differences were observed in the prevalence of favoring polices that limit the number of stores that sell tobacco near schools among race and ethnicity groups, age, marital status groups, perceptions of the seriousness of smoking and tobacco use in their community, and awareness of efforts in the community to decrease tobacco use and prevent secondhand smoke exposure. 

### 3.2. Multivariate, Multinomial Logistic Regression

Table 3 and Table 4 show the results from the multivariate, multinomial logistic regression. 

#### 3.2.1. Support for Tobacco-free City Property Policies

##### Current Smokers

Among current smokers, females had higher odds of favoring tobacco-free city property policies compared to males (OR 1.58, CI 1.00–2.49), although the association was borderline significant. For each one-point increase in SHS knowledge score, the odds of favoring the policy increased by a factor of 1.34 (CI 1.10–1.63). None of the covariates were statistically significantly associated with “neither favoring nor opposing the policy”.

##### Former Smokers

Among former smokers, females had higher odds of favoring tobacco-free city property policies compared to males (OR 2.34, CI 1.36–4.01); and for each one-point increase in SHS knowledge score, the odds of favoring the policy increased by a factor of 1.51 (CI 1.24–1.85). Rural residents had lower odds of favoring the policy than urban residents (OR 0.51, CI 0.29–0.90). None of the covariates were significantly associated with “neither favoring nor opposing” the policy.

##### Never Smokers

Among never smokers, odds of favoring tobacco-free city property policies were higher among females than males (OR 1.86, CI 1.28–2.71); and those with some college education versus those with high school education or less (OR 1.55, CI 1.05–2.27). For every one-point increase in SHS knowledge score, the odds of favoring the policy increased by a factor of 1.44 (CI 1.23–1.68). Never smokers that reported that “any” of the people important to them used tobacco had lower odds of favoring the policy than those that reported “none” of the people important to them used tobacco (OR 0.65, CI 0.44–0.96). None of the covariates were significantly associated with “neither favoring nor opposing” the policy.

#### 3.2.2. Support for Policies that Limit the Number of Stores that Sell Tobacco Near Schools

##### Current Smokers

Among current smokers, the only statistically significant association with favoring policies that limit the number of stores that sell tobacco near schools was for SHS knowledge. For every one-point increase in SHS knowledge score, the odds of favoring the policy increased by a factor of 1.39 (CI 1.15–1.68). None of the covariates were associated with “neither favoring nor opposing” the policy.

##### Former Smokers

Among former smokers, the only statistically significant association with favoring policies that limit the number of stores that sell tobacco near schools was for SHS knowledge. For every one-point increase in SHS knowledge score, the odds of favoring the policy increased by a factor of 1.54 (CI 1.29–1.85). 

Odds of “neither favoring nor opposing” policies that limit the number of stores that sell tobacco near schools were higher among females than males (OR 1.93, CI 1.05–3.56); among those that reported that “any” of the people important to them used tobacco versus former smokers that reported “none” of the people important to them used tobacco (OR 2.11, CI 1.05–4.25); and among those with some college than those with a high school education or less (OR 1.95, CI 1.05–3.64). Former smokers that perceive smoking and tobacco use as a “serious problem” in their community had lower odds of reporting that they “neither favored nor opposed” the policy than former smokers who perceive smoking and tobacco use as “not a serious, only a little serious, or a fairly serious problem in their community” (OR 0.49, CI 0.27, 0.91).

##### Never Smokers

Among never smokers, for every one-point increase in SHS knowledge score, the odds of favoring the policy increased by a factor of 1.38 (CI 1.21, 1.57). The odds of favoring policies that limit the number of stores that sell tobacco near schools were higher among females than males (OR 1.74, CI 1.28, 2.36); and among those that perceived smoking and tobacco use to be a “very serious problem” in their community than those who perceive smoking and tobacco use as “not a serious, only a little serious, or a fairly serious problem” in their community (OR 1.41, CI 1.04, 1.91). Odds of favoring policies that limit the number of stores that sell tobacco near schools were lower among rural residents than urban residents (OR 0.69, CI 0.51, 0.94); and the association was borderline significant among those aged 55+ compared to those aged 18 to 34 years (OR 0.68, CI 0.46–1.00). Those aged 55+ also had lower odds of reporting that they “neither favored nor opposed” the policy than those aged 18 to 34 years (OR 0.46, CI 0.26–0.82). 

See Table S1 for descriptive statistics of all study variables.

## 4. Discussion

Data from the TSET HLP Community Survey show that three out of four (75%) Oklahomans favor policies that establish city property as tobacco-free and two out of three (66%) favor policies that would limit the sale of tobacco near schools. Greater levels of support for tobacco-free city properties, as compared to limiting the sale of tobacco near schools, may be influenced by Oklahoma’s conservative leaning ideology and values. Conservative values that favor an individualistic ethos over social or governmental responsibility and free enterprise in this Right to Work state are deeply rooted. According to the Pew Research Center 38% of Oklahomans identify as conservative as compared to 19% identifying as liberal and 37% as moderate. Furthermore, 59% of Oklahomans would rather have smaller government as compared to a bigger government [30]. While there is a growing norm that smoking in areas open to the public is no longer socially acceptable; government policy that may be perceived as impinging on business owners’ rights may be viewed as less desirable. The political ideology of free enterprise often gives rise to the anti-tobacco regulation argument of “government should ‘butt out’ and let the market give consumers what they want” is a sentiment that resonates with many [31]. Indeed, the Tobacco Industry has a long-standing history in Oklahoma of exploiting this value in appealing to and funding business groups such as the Chamber of Commerce, the Oklahoma Restaurant Association, the Oklahoma Grocers Association, and ubiquitous convenience store QuikTrip to fight against attempts to enact tobacco-related legislation in Oklahoma [32,33]. This history and Oklahomans’ conservative roots may result in less favorable views of government policy that could be framed as interference in a business owner’s right to profit by selling a legal product. It is also possible that favoring or opposing tobacco-related policy could be influenced more by beliefs about government intervention than by the merits of the policy itself [31].

Overall, these data are consistent with the Truth Initiative^®^ finding that residents within Tobacco Nation support tobacco-related policy at similar rates to the rest of the Nation [21]. However, level of support differs by policy type, individual smoking status, as well as among subpopulations.

While never smokers were most likely to favor each of the policies, support was also high among former smokers, and support for both policies was greater than 50% among current smokers. This is consistent with other research that shows that non-smokers are more likely to favor tobacco-related policy than current smokers [34]. Current smokers were also significantly more likely to select the neutral response of “neither favoring nor opposing” for both policies. Further research is warranted to explore whether the selection of this response option by smokers is due to social desirability effect or if this group of people represents an important opportunity for education and advocacy in an effort increase policy support.

Knowledge of the harm associated with SHS exposure was a consistent and strong predictor of support for both policies and among current, former, and never smokers, indicating a continued need for education about the adverse effects of exposure to SHS. In addition to our finding that SHS knowledge is a predictor for policy support, the Centers for Disease Control and Prevention identified increasing knowledge of harm related to SHS as a key outcome indicator for tobacco control programs because of its association with taking action to reduce SHS exposure and increased intentions to quit and quitting tobacco [24]. *Best Practices for Comprehensive Tobacco Control* emphasize the need to increase knowledge among groups of people experiencing greater exposure to SHS and disparities in chronic diseases related to tobacco as well as by using mass-reach health communication campaigns [5]. 

With the exception of current smokers’ support for government policies that limit the sale of tobacco near schools, female gender was also a consistent predictor of support for both policy types and across the smoking status continuum. This finding has been reported in other research looking at tobacco-related policy support [34,35]. Additionally, the Pew Research Center found that females are much more likely to support bigger government as compared to men, 58% and 37%, respectively [36]. A recent report requested by the World Health Organization (WHO) Framework Convention on Tobacco Control defines gender as a “social construct referring to the roles, behaviors, activities, attributes and opportunities” that a population attributes to people of various gender identities. It is distinct from biological sex. The WHO posits that “gender-blind” approaches miss key opportunities to define risk as well as develop appropriate interventions. Therefore, future efforts to create and develop policies that seek to reduce the harm of tobacco use should integrate gender responsive actions [37]. Future research is needed to better understand how gender interacts with support for tobacco-related policy.

When considering rural-urban status, research shows that many rural populations experience higher rates of tobacco use and harm associated with tobacco use, and that states with large rural populations are less likely to have strong, protective tobacco-related laws and policies in place [21,38]. As interest grows in the exploration of rural-urban differences in tobacco use within Tobacco Nation, this analysis contributes insight into policy support. In our analysis, prevalence of support for both policies was lower among rural residents than urban residents. Adjusted odds ratios produced through multivariate, multinomial analysis indicate that rural-urban status remained a predictor for former smokers’ support for tobacco-free city properties and for both former and never smokers’ support for limiting the sale of tobacco near schools. Rural-urban status was not a predictor of support among current smokers. 

Tobacco control programs often count on the support of non-smokers when implementing policy initiatives. However, this study found that never smokers who report that important people in their lives use tobacco are less likely to favor the policy of limiting the sale of tobacco near schools than their peers who report that none of the people important to them use tobacco. Further qualitative research is needed to explore the influence of social influence on policy support by smoking status. 

Strengths of this study include a large, representative sample of Oklahomans, inclusion of both landline and cell phones, and the ability to look at a variety of covariates. In addition to traditional sociodemographic characteristics and community readiness, this study also considered questions related to local tobacco control efforts and the influence of people the respondents perceived as important to their lives. The study also occurred within a unique tobacco-related state context which includes high smoking prevalence, near complete preemption, and a large rural population. Limitations of the study include the cross-sectional, self-report nature of the survey. It is also possible that participants’ responses were influenced by social acceptability bias. Additionally, the study sample was limited to non-institutionalized adults residing in in one of the 63 funded counties. Important to future tobacco control policy planning, it cannot be assumed from these data that participants’ support for policies that establish tobacco-free city properties equates support for clean indoor and outdoor air for all properties. However, other data related to Oklahomans’ attitudes toward policy showed that 77% of Oklahomans favor a law making all public places smokefree [39].

## 5. Conclusions

As efforts continue to create comprehensive clean indoor air laws and protect constituents from the harm related to tobacco use, policy makers should consider the clear support for tobacco-related policy, even among current smokers. Other studies have shown that policy support continues to increase after the implementation of protective, tobacco-related policy due to social norm change [40,41,42]. Efforts to educate the public about the harm of tobacco use and SHS exposure should also continue as this study reinforces its important role along with recent research that shows harmful perceptions of tobacco use is decreasing [43]. This study identifies information that may be useful for education related to tobacco harm and advocacy planning, specifically audience segmentation for key messaging to promote the program-defined outcomes of 100% tobacco-free city-owned property policies and policies that limit the sale of tobacco near schools. Mass-reach health communication interventions, a component of comprehensive tobacco control programs, are one way to educate the population [5]. This study underscores the need for mass-reach campaigns to increase the salience of tobacco use as a serious community problem as well as a means to educate the public about its harm. Oklahoma’s *Tobacco Stops With Me* campaign has been an integral part of comprehensive tobacco control in Oklahoma. The campaign should continue its strategy of ensuring media placement in rural locales as well as developing key messages formulated for males, females, and those that identify as part of other gender groups. Media and advocacy education should also craft messages that support the commonly held, and often exploited by the Tobacco Industry, political ideologies of freedom, fairness, and free enterprise [30]. Educational and advocacy interventions should also be tailored to communities’ level of readiness to address tobacco control and prevention [22]. The findings of this study also contribute to knowledge about factors that influence support for policy among residents of a Tobacco Nation state. 

## Figures and Tables

**Table 1:** Support for policies that prohibit tobacco use on city-owned properties by participant characteristics.

Variables	Policies that Prohibit Tobacco Use on City-Owned Properties					
	Favor		Neither Favor nor Oppose		Oppose	
	*n*	Weighted % (95%CI)	*n*	Weighted % (95%CI)	*n*	Weighted % (95%CI)
Gender						
Male	1347	68.82 (66.09–71.56)	210	12.10 (10.09–14.11)	389	19.07 (16.80–21.35)
Female	2012	81.47 (79.32–83.62)	195	7.71 (6.30–9.11)	245	10.82 (9.05–12.60)
Age						
18–34	1008	75.40 (72.29–78.50)	141	10.82 (8.58–13.06)	167	13.78 (11.28–16.28)
35–54	1337	75.55 (72.88–78.23)	162	9.39 (7.62–11.17)	243	15.05 (12.79–17.31)
55 or older	1014	74.50 (71.14–77.85)	102	9.12 (6.70–11.53)	224	16.39 (13.64–19.14)
Race/Ethnicity						
White	2374	75.96 (73.89–78.04)	263	8.75 (7.41–10.10)	433	15.28 (13.50–17.06)
Black or African-American	187	75.93 (68.64–83.21)	21	10.46 (4.77–16.15)	30	13.61 (8.05–19.18)
American Indian or Alaska Native	309	70.29 (64.22–76.36)	42	10.49 (6.08–14.91)	83	19.21 (14.16–24.26)
Hispanic	196	76.26 (70.03–82.49)	32	11.81 (7.20–16.41)	33	11.93 (7.07–16.79)
Multiracial	230	72.45 (65.24–79.67)	35	14.52 (8.44–20.60)	44	13.03 (8.06–18.00)
Other	63	70.89 (58.83–82.95)	12	18.03 (7.00–29.07)	11	11.08 (3.96–18.19)
Education						
High school or less	996	71.68 (68.60–74.75)	136	10.81 (8.66–12.95)	245	17.52 (14.93–20.10)
Some college or more	2363	77.96 (75.96–79.96)	269	9.15 (7.74–10.57)	389	12.88 (11.28–14.48)
Income						
≤$30,000	746	71.10 (67.10–75.09)	106	12.78 (9.72–15.83)	168	16.13 (12.92–19.34)
$30,000–<$45,000	509	78.18 (73.80–82.56)	64	9.04 (6.25–11.83)	80	12.78 (9.09–16.47)
$45,000–<$60,000	514	75.41 (70.93–79.90)	58	9.35 (6.21–12.50)	94	15.23 (11.59–18.88)
$60,000–<$80,000	496	78.24 (73.96–82.52)	55	8.82 (5.83–11.81)	84	12.94 (9.50–16.37)
$80,000–<$100,000	429	78.38 (73.61–83.16)	46	7.55 (4.75–10.36)	70	14.07 (9.87–18.27)
$100,000–<$150,000	387	75.90 (71.11–80.70)	39	7.82 (4.81–10.83)	77	16.28 (12.12–20.44)
≥$150,000	278	70.99 (65.00–76.99)	37	12.51 (7.83–17.19)	61	16.50 (11.71–21.29)
Region						
Northwest	491	69.60 (64.41–74.78)	72	11.95 (8.36–15.53)	109	18.46 (13.97–22.94)
Northeast	702	72.28 (68.36–76.20)	94	10.25 (7.71–12.79)	137	17.47 (14.01–20.93)
Tulsa	469	77.80 (73.68–81.92)	49	8.95 (6.15–11.76)	77	13.25 (9.87–16.62)
Central	610	79.45 (75.92–82.97)	63	9.13 (6.49–11.78)	85	11.42 (8.74–14.10)
Southwest	525	70.54 (65.73–75.34)	66	11.16 (7.74–14.58)	118	18.30 (14.22–22.38)
Southeast	562	75.94 (71.49–80.39)	61	9.29 (6.22–12.37)	108	14.77 (11.08–18.46)
Urban/Rural						
Urban	1079	78.82 (76.13–81.51)	112	9.07 (7.11–11.02)	162	12.11 (10.01–14.21)
Rural	2280	72.21 (69.93–74.49)	293	10.55 (9.01–12.09)	472	17.24 (15.27–19.21)
Marital status						
Married	2032	75.02 (72.71–77.33)	223	8.94 (7.43–10.44)	390	16.05 (14.06–18.03)
Widowed	87	70.53 (59.47–81.59)	13	10.81 (2.72–18.90)	23	18.66 (9.56–27.75)
Divorced or separated	418	72.17 (67.53–76.81)	62	11.44 (8.11–14.77)	100	16.39 (12.61–20.16)
Never married and other	822	78.07 (74.68–81.47)	107	10.59 (8.02–13.16)	121	11.34 (8.77–13.92)
Cigarette smoking status						
Current smoker	460	56.35 (51.68–61.02)	116	17.17 (13.46–20.88)	213	26.48 (22.38–30.58)
Former smoker	739	72.65 (68.87–76.42)	104	9.65 (7.31–11.98)	164	17.71 (14.37–21.04)
Never smoker	2147	81.95 (79.88–84.02)	182	7.71 (6.25–9.18)	254	10.33 (8.70–11.96)
How many of the people that are important to you use tobacco						
Any	2397	73.0 (70.9–75.1)	321	10.5 (9.0–11.9)	516	16.5 (14.7–18.3)
None	956	82.3 (79.3–85.2)	84	8.1 (5.9–10.3)	114	9.6 (7.4–11.8)
Awareness of local efforts ^1^						
Yes	1720	74.69 (72.23–77.16)	212	9.78 (8.08–11.48)	335	15.53 (13.47–17.58)
No/Don’t know	1638	75.84 (73.36–78.33)	193	9.96 (8.19–11.72)	298	14.20 (12.18–16.22)
Tobacco use as a serious problem ^2^						
Not a serious, only a little serious, or fairly serious	1434	71.56 (68.82–74.30)	219	12.38 (10.26–14.49)	326	16.06 (13.90–18.22)
Very serious	1828	78.92 (76.63–81.22)	170	7.50 (6.09–8.91)	283	13.57 (11.59–15.55)
Knowledge of health effects of SHS						
0	196	51.80 (44.99–58.61)	60	19.88 (14.23–25.53)	120	28.31 (22.38–34.25)
1	415	67.83 (62.70–72.95)	68	11.77 (8.17–15.36)	117	20.41 (15.99–24.82)
2	957	75.32 (71.97–78.66)	105	8.72 (6.58–10.87)	179	15.96 (13.07–18.85)
3	1027	80.90 (77.89–83.90)	105	9.54 (7.22–11.87)	118	9.56 (7.38–11.74)
4	732	84.37 (81.06–87.69)	59	5.98 (3.90–8.07)	82	9.64 (6.88–12.41)
Knowledge of health effects of SHS (*mean, 95%CI*)		2.50 (2.45–2.55)		1.99 (1.82–2.16)		1.91 (1.77–2.04)

^1^ Awareness of any local programs, activities, services, or policies to decrease tobacco use or exposure to SHS; ^2^ How serious of a problem is smoking and tobacco use for people in your community.

**Table 2:** Support for policies that limit the number of stores that sell tobacco near schools by participant characteristics.

Variables	Policies that Limit the Number of Stores that Sell Tobacco Near Schools					
	Favor		Neither Favor nor Oppose		Oppose	
	*n*	Weighted % (95%CI)	*n*	Weighted % (95%CI)	*n*	Weighted % (95%CI)
Gender						
Male	1218	60.61 (57.72–63.50)	249	14.16 (12.03–16.29)	486	25.23 (22.67–27.79)
Female	1793	72.70 (70.25–75.15)	275	12.38 (10.52–14.24)	381	14.92 (13.00–16.85)
Age						
18–34	933	68.30 (64.98–71.63)	170	14.47 (11.88–17.07)	220	17.22 (14.56–19.88)
35–54	1199	66.65 (63.67–69.62)	216	13.56 (11.39–15.73)	319	19.79 (17.26–22.33)
55 or older	879	64.28 (60.63–67.93)	138	10.64 (8.28–12.99)	328	25.09 (21.77–28.41)
Race/Ethnicity						
White	2088	65.56 (63.26–67.87)	359	13.16 (11.46–14.85)	625	21.28 (19.30–23.26)
Black or African-American	174	73.19 (65.98–80.39)	22	8.33 (4.05–12.60)	42	18.49 (12.04–24.93)
American Indian or Alaska Native	293	66.36 (60.00–72.71)	54	14.81 (9.60–20.02)	86	18.83 (13.84–23.83)
Hispanic	187	71.38 (64.72–78.03)	36	12.40 (7.83–16.97)	39	16.23 (10.59–21.87)
Multiracial	208	63.41 (55.48–71.35)	43	20.43 (12.97–27.89)	59	16.16 (11.02–21.30)
Other	61	71.47 (60.01–82.93)	10	12.08 (3.97–20.19)	16	16.45 (7.03–25.87)
Education						
High school or less	912	64.74 (61.50–67.99)	159	13.12 (10.77–15.48)	308	22.13 (19.35–24.92)
Some college or more	2099	68.25 (65.97–70.54)	365	13.36 (11.63–15.08)	559	18.39 (16.50–20.28)
Income						
≤$30,000	713	65.50 (61.36–69.64)	100	12.83 (9.75–15.91)	206	21.67 (18.12–25.22)
$30,000–<$45,000	449	70.67 (65.83–75.50)	87	13.35 (9.70–16.99)	118	15.99 (12.21–19.76)
$45,000–<$60,000	456	65.52 (60.43–70.61)	91	15.61 (11.51–19.72)	123	18.87 (14.74–23.00)
$60,000–<$80,000	453	73.44 (68.88–78.00)	83	13.37 (9.80–16.93)	95	13.20 (9.79–16.61)
$80,000–<$100,000	388	69.14 (63.79–74.49)	51	8.47 (5.44–11.49)	110	22.40 (17.45–27.34)
$100,000–<$150,000	319	61.18 (55.69–66.68)	68	14.60 (10.54–18.67)	115	24.21 (19.32–29.11)
≥$150,000	233	60.50 (54.24–66.76)	44	14.06 (9.37–18.75)	100	25.44 (19.92–30.95)
Region						
Northwest	431	56.93 (51.38–62.49)	91	17.79 (13.13–22.44)	153	25.28 (20.33–30.23)
Northeast	638	63.59 (59.37–67.80)	111	13.55 (10.46–16.64)	186	22.86 (19.10–26.62)
Tulsa	417	68.73 (64.12–73.35)	67	11.99 (8.64–15.35)	111	19.27 (15.41–23.13)
Central	556	72.79 (68.95–76.63)	82	12.00 (9.14–14.87)	116	15.20 (12.15–18.25)
Southwest	476	62.51 (57.44–67.58)	83	14.78 (10.77–18.80)	149	22.71 (18.37–27.05)
Southeast	493	67.13 (62.37–71.88)	90	12.32 (9.20–15.43)	152	20.55 (16.35–24.76)
Urban/Rural						
Urban	973	71.25 (68.29–74.21)	149	12.00 (9.81–14.19)	227	16.75 (14.35–19.14)
Rural	2038	62.89 (60.45–65.33)	375	14.33 (12.50–16.16)	640	22.78 (20.63–24.94)
Marital status						
Married	1818	67.57 (65.10–70.04)	306	12.35 (10.55–14.14)	520	20.08 (17.98–22.18)
Widowed	80	56.67 (44.29–69.04)	10	12.06 (3.69–20.43)	35	31.28 (19.30–43.25)
Divorced or separated	367	61.22 (56.12–66.31)	86	16.19 (12.40–19.99)	125	22.59 (18.22–26.96)
Never married and other	746	69.49 (65.73–73.26)	122	13.32 (10.44–16.20)	187	17.18 (14.15–20.22)
Cigarette smoking status						
Current smoker	450	54.37 (49.70–59.04)	123	17.93 (14.24–21.62)	221	27.69 (23.49–31.90)
Former smoker	638	60.98 (56.82–65.14)	133	14.50 (11.29–17.70)	242	24.52 (20.91–28.13)
Never smoker	1910	72.67 (70.31–75.04)	267	11.49 (9.77–13.21)	399	15.83 (13.90–17.77)
How many of the people that are important to you use tobacco						
Any	2165	65.0 (62.7–67.3)	415	14.3 (12.6–16.0)	659	20.7 (18.8–22.6)
None	839	72.1 (68.6–75.6)	108	10.0 (7.6–12.4)	205	17.9 (14.9–20.9)
Awareness of local efforts ^1^						
Yes	1515	64.63 (61.92–67.33)	288	13.70 (11.70–15.70)	471	21.68 (19.35–24.00)
No/Don’t know	1495	68.83 (66.14–71.52)	236	12.84 (10.85–14.84)	395	18.33 (16.10–20.56)
Tobacco use as a serious problem ^2^						
Not a serious, only a little serious, or fairly serious	1290	64.08 (61.17–66.99)	275	14.32 (12.18–16.47)	414	21.60 (19.08–24.12)
Very serious	1636	69.38 (66.79–71.97)	226	11.92 (10.01–13.83)	421	18.70 (16.55–20.85)
Knowledge of health effects of SHS						
0	165	41.49 (34.76–48.21)	74	21.19 (15.66–26.72)	138	37.33 (30.76–43.89)
1	369	56.89 (51.40–62.37)	98	19.81 (15.14–24.47)	135	23.30 (18.58–28.02)
2	878	68.42 (64.88–71.95)	130	11.86 (9.36–14.36)	233	19.72 (16.70–22.74)
3	890	69.93 (66.42–73.45)	139	12.67 (10.05–15.30)	222	17.39 (14.52–20.26)
4	677	79.54 (75.97–83.12)	75	7.84 (5.39–10.30)	119	12.61 (9.73–15.50)
Knowledge of health effects of SHS (*mean, 95% CI*)		2.54 (2.48–2.60)		2.01 (1.87–2.15)		2.00 (1.89–2.12)

^1^ Awareness of any local programs, activities, services, or policies to decrease tobacco use or exposure to SHS; ^2^ How serious of a problem is smoking and tobacco use for people in your community.

**Table 3:** Association of support for policies that prohibit tobacco use on city-owned properties with other study variables, by smoking status.

Variables		Adjusted OR (95%CI) ^1^					
		Current Smoker		Former Smoker		Never Smoker	
	Category	Favor	Neither Favor nor Oppose	Favor	Neither Favor nor Oppose	Favor	Neither Favor nor Oppose
Gender	Female vs. male	1.580 (1.004, 2.486)	1.087 (0.577, 2.046)	2.338 (1.362, 4.012)	1.129 (0.573, 2.227)	1.858 (1.275, 2.708)	0.989 (0.575, 1.702)
Region	Rural vs. urban	1.053 (0.635, 1.747)	0.685 (0.355, 1.320)	0.510 (0.288, 0.902)	0.950 (0.430, 2.100)	0.781 (0.542, 1.125)	0.965 (0.567, 1.641)
People that are important to you use tobacco	Any vs. none	1.041 (0.312, 3.476)	0.536 (0.150, 1.914)	0.724 (0.409, 1.280)	1.384 (0.572, 3.349)	0.654 (0.444, 0.963)	0.703 (0.398, 1.242)
Awareness of local efforts ^2^	Yes vs. no/don’t know	1.174 (0.727, 1.895)	1.230 (0.658, 2.300)	0.732 (0.447, 1.198)	0.535 (0.271, 1.054)	1.057 (0.732, 1.526)	1.027 (0.604, 1.746)
Knowledge of health effects of SHS		1.339 (1.103, 1.626)	1.146 (0.890, 1.476)	1.514 (1.238, 1.852)	0.857 (0.647, 1.137)	1.437 (1.226, 1.684)	1.038 (0.825, 1.305)
Age	35–54 vs. 18–34	1.239 (0.711, 2.161)	0.681 (0.333, 1.394)	1.713 (0.885, 3.315)	1.304 (0.543, 3.134)	0.848 (0.555, 1.295)	0.749 (0.427, 1.313)
	≥55 vs. 18–34	0.727 (0.390, 1.355)	0.660 (0.287, 1.522)	1.659 (0.895, 3.076)	1.155 (0.489, 2.729)	1.063 (0.676, 1.671)	0.624 (0.300, 1.298)
Education	Some college or more vs. high school or less	0.922 (0.590, 1.441)	0.894 (0.490, 1.630)	0.941 (0.571, 1.552)	1.270 (0.648, 2.489)	1.545 (1.051, 2.272)	1.458 (0.834, 2.548)
Tobacco use as a serious problem ^3^	Very serious vs. not a serious, only a little serious, or fairly serious	1.537 (0.966, 2.445)	0.652 (0.352, 1.207)	1.356 (0.810, 2.271)	0.789 (0.400, 1.556)	1.306 (0.895, 1.907)	0.618 (0.362, 1.056)

^1^ Oppose is the reference category; ^2^ Awareness of any local programs, activities, services, or policies to decrease tobacco use or exposure to SHS; ^3^ How serious of a problem is smoking and tobacco use for people in your community.

**Table 4:** Association of support for policies that limit the number of stores that sell tobacco near schools with other study variables, by smoking status.

Variables		Adjusted OR (95%CI) ^1^					
		Current Smoker		Former Smoker		Never Smoker	
	Category	Favor	Neither Favor nor Oppose	Favor	Neither Favor nor Oppose	Favor	Neither Favor nor Oppose
Gender	Female vs. male	1.336 (0.855, 2.087)	1.492 (0.825, 2.698)	2.306 (1.511, 3.518)	1.934 (1.05, 3.562)	1.736 (1.278, 2.36)	1.262 (0.822, 1.936)
Region	Rural vs. urban	1.196 (0.734, 1.948)	0.958 (0.513, 1.787)	0.447 (0.282, 0.709)	0.836 (0.426, 1.639)	0.689 (0.507, 0.936)	0.866 (0.566, 1.327)
People that are important to you use tobacco	Any vs. none	1.620 (0.562, 4.676)	1.638 (0.478, 5.609)	1.034 (0.644, 1.660)	2.111 (1.049, 4.247)	0.913 (0.661, 1.260)	1.166 (0.739, 1.842)
Awareness of local efforts ^2^	Yes vs. no/don’t know	1.036 (0.644, 1.667)	1.014 (0.553, 1.857)	0.674 (0.437, 1.039)	0.813 (0.441, 1.499)	0.807 (0.597, 1.090)	0.893 (0.583, 1.368)
Knowledge of Health effects of SHS		1.39 (1.147, 1.684)	0.948 (0.744, 1.208)	1.542 (1.287, 1.847)	1.058 (0.835, 1.341)	1.377 (1.207, 1.571)	0.983 (0.819, 1.179)
Age	35–54 vs. 18–34	0.658 (0.375, 1.156)	0.575 (0.283, 1.171)	1.477 (0.844, 2.587)	0.868 (0.401, 1.878)	1.060 (0.746, 1.508)	1.011 (0.631, 1.622)
	≥55 vs. 18–34	0.877 (0.474, 1.623)	0.479 (0.211, 1.089)	1.120 (0.638, 1.965)	0.614 (0.282, 1.336)	0.677 (0.460, 0.998)	0.461 (0.259, 0.821)
Education	Some college or more vs. high school or less	1.015 (0.658, 1.564)	1.041 (0.584, 1.856)	1.320 (0.858, 2.031)	1.953 (1.048, 3.641)	0.896 (0.647, 1.243)	1.110 (0.706, 1.748)
Tobacco use as a serious problem ^3^	Very serious vs. not a serious, only a little serious, or fairly serious	1.214 (0.756, 1.950)	1.030 (0.557, 1.905)	1.018 (0.662, 1.566)	0.494 (0.267, 0.913)	1.413 (1.043, 1.914)	1.261 (0.823, 1.934)

^1^ Oppose is the reference category; ^2^ Awareness of any local programs, activities, services, or policies to decrease tobacco use or exposure to SHS; ^3^ How serious of a problem is smoking and tobacco use for people in your community.

## Supplementary Materials

The following are available online at https://www.mdpi.com/1660-4601/16/18/3378/s1, Table S1: Descriptive statistics of study variables.
