# Supplementary material for: Support for Local Tobacco Policy in a Preemptive State
Source: Int J Environ Res Public Health. 2019 Sep 12;16(18):3378. doi: 10.3390/ijerph16183378 (PMC6766053; doi:10.3390/ijerph16183378)
Supplement: Supplementary file 1 [file ijerph-16-03378-s001.pdf]

Table S1. Descriptive statistics of study variables

| Variables | Overall n | Overall Weighted % (95%CI) |
|---|---|---|
| Gender | | |
| Male | 1980 | 49.34 (47.35, 51.32) |
| Female | 2481 | 50.66 (48.68, 52.65) |
| Age | | |
| 18-34 | 1334 | 37.33 (35.36, 39.30) |
| 35-54 | 1763 | 40.46 (38.51, 42.41) |
| 55 or older | 1364 | 22.21 (20.70, 23.71) |
| Race/Ethnicity | | |
| White | 3113 | 67.96 (66.08, 69.85) |
| Black or African-American | 242 | 7.29 (6.18, 8.41) |
| American Indian or Alaska Native | 441 | 6.81 (5.95, 7.67) |
| Hispanic | 265 | 9.44 (8.14, 10.75) |
| Multiracial | 313 | 5.98 (5.05, 6.90) |
| Other | 87 | 2.51 (1.87, 3.15) |
| Education | | |
| High school or less | 1409 | 43.23 (41.21, 45.25) |
| Some college or more | 3052 | 56.77 (54.75, 58.79) |
| Income | | |
| ≤ $30,000 | 1042 | 21.58 (19.97, 23.19) |
| $30,000 – <$45,000 | 664 | 14.16 (12.75, 15.58) |
| $45,000 – <$60,000 | 673 | 13.29 (11.99, 14.59) |
| $60,000 – <$80,000 | 640 | 15.52 (14.04, 16.99) |
| $80,000 – <$100,000 | 552 | 10.95 (9.76, 12.13) |
| $100,000 – <$150,000 | 510 | 14.51 (13.05, 15.98) |
| ≥ $150,000 | 380 | 9.97 (8.79, 11.18) |

| Variables | Overall | |
|---|---|---|
| | n | Weighted % (95%CI) |
| Region | | |
| Northwest | 684 | 10.11 (9.33, 10.90) |
| Northeast | 946 | 20.96 (19.67, 22.25) |
| Tulsa | 600 | 17.36 (16.07, 18.65) |
| Central | 764 | 28.55 (26.91, 30.20) |
| Southwest | 716 | 11.13 (10.21, 12.04) |
| Southeast | 751 | 11.89 (11.01, 12.76) |
| Urban/Rural | | |
| Urban | 1364 | 45.91 (44.22, 47.61) |
| Rural | 3097 | 54.09 (52.39, 55.78) |
| Marital status | | |
| Married | 2677 | 52.77 (50.77, 54.76) |
| Widowed | 127 | 2.67 (2.03, 3.31) |
| Divorced or separated | 592 | 16.98 (15.40, 18.56) |
| Never married and other | 1065 | 27.58 (25.74, 29.42) |
| Cigarette smoking status | | |
| Current smoker | 805 | 18.32 (16.79, 19.86) |
| Former smoker | 1027 | 21.31 (19.72, 22.91) |
| Never smoker | 2610 | 60.36 (58.42, 62.30) |
| How many of the people that are important to you use tobacco | | |
| None | 1171 | 24.84 (23.16, 26.52) |
| Any | 3279 | 75.16 (73.48, 76.84) |
| How serious of a problem is smoking and tobacco use for people in your community | | |
| Not a serious, only a little serious, or fairly serious | 2005 | 45.93 (43.91, 47.95) |

| Variables | Overall | |
| --- | --- | --- |
| | n | Weighted % (95%CI) |
| Very serious | 2314 | 54.07 (52.05, 56.09) |
| Awareness of any programs, activities, services, or policies to decrease tobacco use or exposure to SHS | | |
| Yes | 2296 | 49.00 (47.01, 50.99) |
| No/Don't know | 2163 | 51.00 (49.01, 52.99) |
| Knowledge of health effects of SHS | | |
| 0 | 386 | 8.94 (7.79, 10.09) |
| 1 | 610 | 14.56 (13.10, 16.01) |
| 2 | 1260 | 28.19 (26.39, 29.99) |
| 3 | 1262 | 28.30 (26.50, 30.11) |
| 4 | 882 | 20.01 (18.41, 21.60) |
| Knowledge of health effects of SHS (*mean, 95%CI)* | | 2.36 (2.31, 2.41) |
